# Fc engineering of anti-Nectin-2 antibody improved thrombocytopenic adverse event in monkey

**DOI:** 10.1371/journal.pone.0196422

**Published:** 2018-05-03

**Authors:** Tsutomu Oshima, Hideaki Miyashita, Yoshimasa Ishimura, Yuki Ito, Yoko Tanaka, Akira Hori, Toshio Kokubo, Tomofumi Kurokawa

**Affiliations:** 1 Immunobiologics, Takeda California Inc., San Diego, California, United States of America; 2 Pharmaceutical Sciences, Takeda Pharmaceutical Company Limited, Hikari, Yamaguchi, Japan; 3 Pharmaceutical Research Division, Takeda Pharmaceutical Company Limited, Fujisawa, Kanagawa, Japan; Duke University School of Medicine, UNITED STATES

## Abstract

Nectin-2 is a transmembrane glycoprotein which is involved in the process of Ca^2+^-independent cell-cell adhesion. In our previous study, we have demonstrated that Nectin-2 is over-expressed in breast and ovarian cancer tissues by using gene expression analysis and immunohistochemistry. Furthermore, we discovered multiple anti-Nectin-2 fully human monoclonal antibodies which inhibited tumor growth in *in vivo* subcutaneous xenograft models with antibody-dependent cellular cytotoxicity (ADCC) as the principal mechanism of action. In this report, we assessed the toxicity of Y-443, a fully human IgG_1_/kappa anti-Nectin-2 monoclonal antibody exhibiting strong *in vitro* ADCC and *in vivo* anti-tumor activity in cynomolgus monkeys (*Macaca fascicularis* (Cynos)). Unexpectedly, upon administration, Y-443 induced strong thrombocytopenia through Nectin-2 expressed on Cyno platelets, presumably followed by phagocytosis in the mononuclear phagocytic system. To mitigate the adverse safety profile, we mutated the Fc region of Y-443 to reduce the Fc binding activity to Fcγ receptor I, which is the primary receptor for phagocytosis on macrophages. Moreover, we further engineered the Fc through defucosylation to maintain ADCC activity. The resultant Fc engineered antibody, termed Y-634, demonstrated diminished thrombocytopenia in Cyno toxicological studies and maintained anti-tumor activity in a mouse xenograft model. These findings suggest that Y-634 may have a therapeutic potential for the treatment of Nectin-2 positive cancers, and moreover, Fc engineering is a potential mitigation strategy to ameliorate safety liabilities in antibody induced thrombocytopenia while maintaining antibody potency.

## Introduction

Nectin-2, a single-pass type I transmembrane glycoprotein with an extracellular region consisting of three immunoglobulin (Ig) -like domains, is a plasma membrane component of adherens junctions that mediates Ca^2+^-independent cell-cell adhesion [1]. Previous studies reported that the second Ig-like domain is important for the formation of Nectin-2-Nectin-2 homo-*cis*-dimers [1–8]. The Nectin-2 homo-*cis*-dimer is able to interact with other Nectin-2 homo-*cis*-dimer or Nectin-3 homo-*cis*-dimer expressed on other cells to form homo-*trans*-dimers or hetero-*trans*-dimers, respectively, via the first Ig-like domain in Nectin-2 or Nectin-3 [1–8]. Those homo- and hetero-*trans*-dimers then induce other cell-cell adhesion event via other cell adhesion molecules such as E-cadherin and claudin, to form tight junction [9–12]. Nectin-2 also plays a role as organizer of Sertoli cell-spermatid junctions and synapse formation in testes and neurons, respectively [13–15]. Furthermore, in addition of cell adhesion functions, Nectin-2 mediates both entry and spreading of infection from various viruses [16] and has also been reported that DNAM-1 (CD226) and TIGIT interact Nectin-2 to activate immune cells [17–20].

In a previous study, we reported that Nectin-2 is over-expressed in breast and ovarian cancer and is involved in cancer proliferation [21]. We generated numerous Nectin-2-specific fully human monoclonal antibodies (mAbs) and demonstrated the anti-tumor effect of the selected mAb clone, Y-443 (human IgG_1_/kappa), on breast cancer and ovarian cancer cells [21]. We also performed *in vitro* and *in vivo* studies of Y-443 and Y-443 IgG_4_ (where the constant region is substituted to human IgG_4_ isotype) and found that the main mechanism of action appeared to be antibody-dependent cellular cytotoxicity (ADCC) [21].

The toxicological studies have been conducted in cynomolgus monkeys (*Macaca fascicularis* (Cynos)) since Y-443 did not cross-react to mouse Nectin-2, but cross-reacted Cyno Nectin-2 with affinity nearly identical to human Nectin-2. While the anti-tumor effect was promising, the anti-Nectin-2 mAb exhibited unexpected “idiopathic thrombocytopenic purpura (ITP)-like” thrombocytopenia in Cynos. ITP is an immune-mediated bleeding disease chiefly caused by autoantibodies directed against membrane-bound targets including GPIIb/IIIa and GPIb-IX on platelets [22–24], resulting in phagocytosis in the mononuclear phagocytic system (MPS). Conversely, it has been reported that other platelet antigens can cause thrombocytopenia via alternative mechanism of actions [25, 26]. In addition to autoantibodies, it has also been reported that therapeutic antibodies such as anti-TNFα (infliximab), anti-CD11a (efalizumab), and anti-CD20 (rituximab) antibodies occasionally trigger platelet-specific autoantibodies and induce thrombocytopenia; however the mechanism of action remained unclear [27]. Previous reports have shown that the clearance of platelets coated with IgG autoantibodies is accelerated by phagocytosis through Fcγ receptors (FcγRs) expressed on tissue macrophages in the reticuloendothelial system, particularly in the spleen [22, 23, 28]. Wallace *et al*. suggested that FcγRI is the most relevant to phagocytosis of antibody-bound platelets by macrophages [29], and intravenous infusion of gammaglobulin (IVIg) and Fcγ fragments has been used clinically for the treatment of such ITP to prevent the phagocytic events [30, 31].

Therapeutic antibodies are predominantly comprised of human IgG_1_ and can elicit immune effector functions via the engagement of both humoral and cell-mediated immunity through interaction with the Fc portion of the antibody. It has been reported that Leu^234^-Gly^237^ of human IgG_1_, particularly Leu^235^ is a critical amino acid for mediating the Fc-FcγRI interaction [32–37]. In this paper, we report that the anti-Nectin-2 antibody Y-443 induces strong thrombocytopenia in Cynos. We further present the ability of Fc engineering, including Leu^235^-substitution and defucosylation of Y-443, to ameliorate thrombocytopenia through decreased binding to FcγRI while presenting ADCC that is essential for its pharmacological activity.

## Materials and methods

### Cells

MDA-MB-231 cells were purchased from the American Type Culture Collection. MDA-MB-231 cells were grown in Leibovitz’s L-15 medium containing 10% fetal bovine serum in a humidified incubator at 37°C.

### Preparation of recombinant proteins

Recombinant extracellular domains (ED) of human or Cyno Nectin-2 fused with FLAG (Nectin-2-ED-FLAG) or human Fc (Nectin-2-ED-Fc) at its C-terminus were prepared as described previously [21]. Recombinant human FcγRI and FcγRIIa were purchased from R&D systems. Recombinant human FcγRIIb and FcγRIIIa (158F) were prepared in-house. Briefly, a eukaryotic expression vector pcDNA3.1 in which a cDNA encoding a sequence of extracellular domain of FcγR with a 6 histidine-tag at its C-terminus was inserted was constructed, and it was transiently expressed in FreeStyle 293-F cells by FreeStyle^TM^ 293 Expression System (Invitrogen). The recombinant proteins were purified from the culture supernatant by Ni-NTA Agarose affinity chromatography.

### Production and Fc modification of anti-Nectin-2 mAbs

Anti-Nectin-2 fully human mAb, Y-443, was generated by immunizing transchromosomic fully human antibody producing mice carrying the complete locus for the human immunoglobulin heavy chain gene and a transgene for the human immunoglobulin kappa light chain (Kyowa Hakko Kirin Co., Ltd.) with recombinant Nectin-2 protein as described previously [21]. The cDNAs encoding the variable regions of heavy chain and light chain of the antibody were isolated from the hybridoma, and were inserted into the GS expression vector pEE6.4 (Lonza Biologics) with human IgG_1_ constant region and pEE12.4 (Lonza Biologics) with human kappa constant region, respectively. The two vectors were combined to obtain a double-gene vector carrying the heavy and light chain according to the manufacturer's guidelines. The expression vector was transfected into CHOK1SV cells, and a stable transfectant was obtained according to manufacturer's standard protocols. The stable transfectant was expanded in CD CHO medium (Invitrogen) containing 25 mM methionine sulfoximine, and the recombinant Y-443 antibody was purified from the culture supernatant by recombinant protein A chromatography (MabSelect SuRe, GE Healthcare), followed by ion-exchange chromatography using Capto Q and Capto S columns (GE Healthcare). The purified Y-443 was concentrated and buffer-exchanged in Dulbecco’s phosphate buffered saline. Endotoxin removal was performed using ActiClean Etox resin (Sterogene Bioseparations) according to manufactures guidelines.

To generate Fc mutants, a single amino acid mutation to aspartic acid (Asp, D) or tyrosine (Tyr, Y) was introduced at leucine (Leu, L) position 235 (Kabat numbering) in the heavy chain gene of Y-443 using QuikChange Lightning Site-Directed Mutagenesis Kit (Stratagene) with corresponding primers and the double gene vector encoding Y-443 as a template. The Fc mutant vectors were transiently transfected to FreeStyle 293-F cells using the FreeStyle^TM^ 293 Expression System. The recombinant Fc variant antibodies were purified from the culture supernatant by Protein A chromatography, followed by size-exclusion chromatography and membrane filtration.

The recombinant defucosylated Y-443 (L235D) antibody, termed Y-634, was prepared by transfection of Y-443 (L235D) plasmid into fucosyltransferase knockout CHO cells (POTELLIGENT^®^ Cells, BioWa, Inc.) and stable transfectants were generated. One of the stable transfectants, #90–4, was further cultured in 90% CD CHO, 10% CD DG44 (Invitrogen) medium, and the expressed Y-634 was purified from the culture supernatant as described in aforementioned methods.

### Analysis of recombinant anti-Nectin-2 mAbs

The purity of the recombinant antibodies was tested by SDS-PAGE and a size-exclusion chromatography using Superdex 200 10/300 GL (GE Healthcare). Endotoxin content was measured by Endospecy ES-24S Set (Seikagaku Co.). L-fucose content of the antibodies was determined by the carbohydrate constituent analysis [38].

### Binding activity of anti-Nectin-2 mAbs to human and Cyno Nectin-2 or human FcγRs

Antigen binding affinity of the anti-Nectin-2 mAbs was evaluated by a kinetic measurement with a BIACORE 2000 using recombinant human and Cyno Nectin-2-ED-Fc proteins, and the equilibrium dissociation constant (K_D_) was determined using BIAevaluation software. The binding of Y-443 and the Fc engineered antibodies against human FcγRs was measured by an ELISA using immunoplates coated with recombinant human FcγRI, FcγRIIa, FcγRIIb, and FcγRIIIa (158F). Various concentrations of antibodies were applied to the plates, and the bound antibody was detected by HRP-labeled anti-human IgG (H+L) antibody (Immuno-Biological Labolatories Co., Ltd). The EC_50_ was determined by GraphPad Prism (GraphPad Software, Inc.).

### Pharmacological studies in mice

The anti-tumor effect of anti-Nectin-2 mAbs was evaluated in a mouse subcutaneous xenograft model. C.B17/Icr-scid/scid Jcl mice (6-week old, female) were purchased from CLEA Japan. Mice were housed in groups of 5 per plastic cage and were acclimated for at least 7 days prior to study start. Room temperature was maintained at 24±1°C with a 12/12 hour light/dark cycle. Food and water were available *ad libitum*. A 100 μL volume of MDA-MB-231 breast cancer cells (3 x 10^6^ cells) and Matrigel (Becton Dickinson) was subcutaneously inoculated into the left flank of mice. When the tumor volume reached to around 120–200 mm^3^, the mice were randomly grouped. In the first experiment, dose escalating Y-443 (0.1, 0.3, 1, 3 and 10 mg/kg/10 mL) and vehicle (Dulbecco’s phosphate buffered saline (-)) were intravenously injected to 5 mice in each treatment group once a week for 3 weeks under physical restraint. Tumor diameter was measured with a calliper twice per week and the approximate tumor volume was calculated by the equation of 0.5 x length x (width)^2^ during the treatment period to monitor tumor growth. In the second experiment, the anti-tumor effect of Y-634 (0.1, 0.3 and 1 mg/kg/10 mL) was compared to that of Y-443 (0.3 mg/kg/10 mL) using the methodology described in the first experimental group. Statistical analysis was performed by the Williams’ test.

### Housing of Cynos

All of Cynos (*Macaca fascicularis* at 3 years and 2 months to 5 years and 8 months of age, Nafovanny and/or Siconbrec) were individually housed in metal cages set on racks in an animal room based on the protocol of toxicological study approved by the Committee on the Ethics of Animal Experiments of Takeda Pharmaceutical Company Limited. Each monkey was fed 150 g of a pelleted diet (Certified primate diet #5048, PMI Feeds Inc.) once-daily. The Cynos were allowed free access to tap water.

### Toxicological studies in Cynos

Single dose of Y-443 (1, 3, 10 and 50 mg/kg) and vehicle (25 mM sodium acetate, 125 mM NaCl, pH 5.5) were intravenously injected (2 mL/minute) to 2 male and 2 female monkeys (Nafovanny and Siconbrec) in each group followed by 2 weeks observation period. Single dose of Y-634 (10 mg/kg) was also intravenously injected to 2 male monkeys (Siconbrec) followed by 2-week observation period. Y-634 (3 and 10 mg/kg/week) and vehicle (Dulbecco’s phosphate buffered saline (-)) were intravenously injected for 3 weeks to 2 female monkeys (Nafovanny and Siconbrec) in each group followed by 2-week observation period. Clinical signs, body weights, food consumption, bleeding time, hematology, coagulation test, gross pathology, organ weights, histopathology and/or toxicokinetics were examined. Blood samples for hematology were collected from femoral vein of each animal using a syringe with sodium heparin and were transferred to tubes containing EDTA-2K under physical restraint. Blood samples for coagulation test were collected from femoral vein with a syringe containing 0.1 mL of 3.13% sodium citrate and were centrifuged at 1500×g for 10 minutes to obtain plasma. The values of hematology were determined or calculated with an automated hematology analyzer (ADVIA120, Siemens Healthcare Diagnostics K.K.) and the values for coagulation test were determined with an automated blood coagulation analyzer (CA-1000, Sysmex Corporation). F gross pathology, organ weights and histopathology, all of Cynos were anesthetized by intramuscular injection of ketamine hydrochloride and were euthanized by exsanguinations from the common carotid artery at necropsy. Systemic organs for histopathology were fixed in 10 volume percent (vol%) neutral buffered formalin. The eyes and testes were fixed in 2.5 weight/volume percent glutaraldehyde in 10 vol% neutral buffered formalin and Bouin’s fluid, respectively, and then preserved in 10 vol% neutral buffered formalin. Fixed samples were embedded in paraffin, sectioned, stained with hematoxylin and eosin, and examined microscopically. Blood samples for toxicokinetics were collected from the vein of the extremities by syringes and transferred to tubes containing separating agent and were centrifuged at about 1500×g for 15 minutes to obtain plasma and stored in ice.

### Measurement of Y-443 or Y-634 in Cyno serum

Cyno serum samples were applied to immunoplates coated with recombinant human Nectin-2 protein (R&D systems). The bound antibody was detected by HRP-labeled anti-human IgG (H+L) antibody (Immuno-Biological Labolatories Co., Ltd). The serum concentration of Y-443 or Y-634 was calculated from its standard curve.

### ADCC assay

Human peripheral blood mononuclear cells (PBMC) purchased from AllCells, LLC and were cultured in RPMI1640 medium containing 10% fetal bovine serum, 0.1 nM human IL-2 (DIACLONE Research) and 55 μM 2-mercaptoethanol for 24 hours. PBMC were incubated with Calcein AM-labeled MDA-MB-231 cells at a ratio of 50:1 in the presence of anti-Nectin-2 mAb for 4 hours at 37°C. After the incubation, total and dead MDA-MB-231 cell numbers were measured and dead cell ratio was calculated by Acumen Explorer (TTP labtech).

### Ethics statement

All of the animal studies were carried out in strict accordance with the recommendations in the Guide for the Care and Use of Laboratory Animals of the National Institutes of Health. The protocols were approved by the Committee on the Ethics of Animal Experiments of Takeda Pharmaceutical Company Ltd. (Permit Numbers for Cyno studies: TEACUC-A1-012 and TEACUC-A1-014, Permit Number for mouse studies: TEACUC-E1-031).

## Results

### Tumor growth inhibition by Y-443 against MDA-MB-231 in a mouse subcutaneous xenograft model

**Fig 1:**
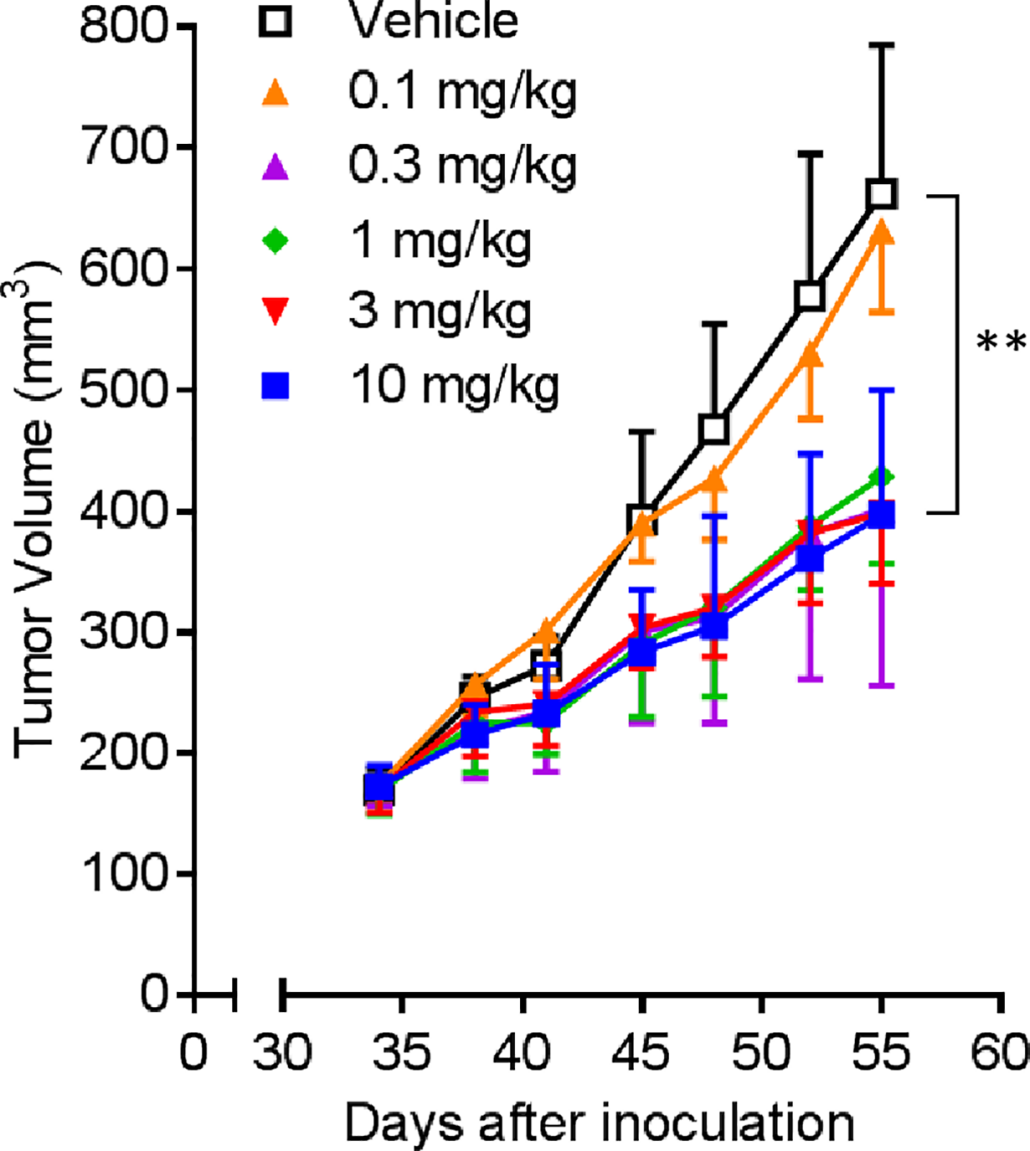
Anti-tumor effect of Y-443 in a mouse subcutaneous xenograft model with MDA-MB-231 breast cancer cells. MDA-MB-231 breast cancer cells were subcutaneously inoculated with Matrigel into a flank of C.B17/Icr-scid/scid mice. On days 34, 41 and 48 after the cell inoculation, Y-443 at a dose of 0.1, 0.3, 1, 3, 10 mg/kg or vehicle was intravenously administered (n = 5). The results are the mean ± S.D. of tumor volume. **: p < 0.001 versus the tumor volume treated with vehicle by one-tailed Williams’ test.

We have previously reported that Y-443, one of the anti-Nectin-2 fully human mAbs, significantly inhibited tumor growth in a MDA-MB-231 mouse subcutaneous xenograft model with ADCC as the central mechanism of action [21]. Here, we evaluated Y-443 in the same MDA-MB-231 mouse subcutaneous xenograft model with lower dose to determine its minimum effective dose. As shown in Fig 1, Y-443 significantly demonstrated potent anti-tumor effect at 0.3 mg/kg, but not at 0.1 mg/kg.

### Adverse effects of Y-443 in Cynos

**Fig 2:**
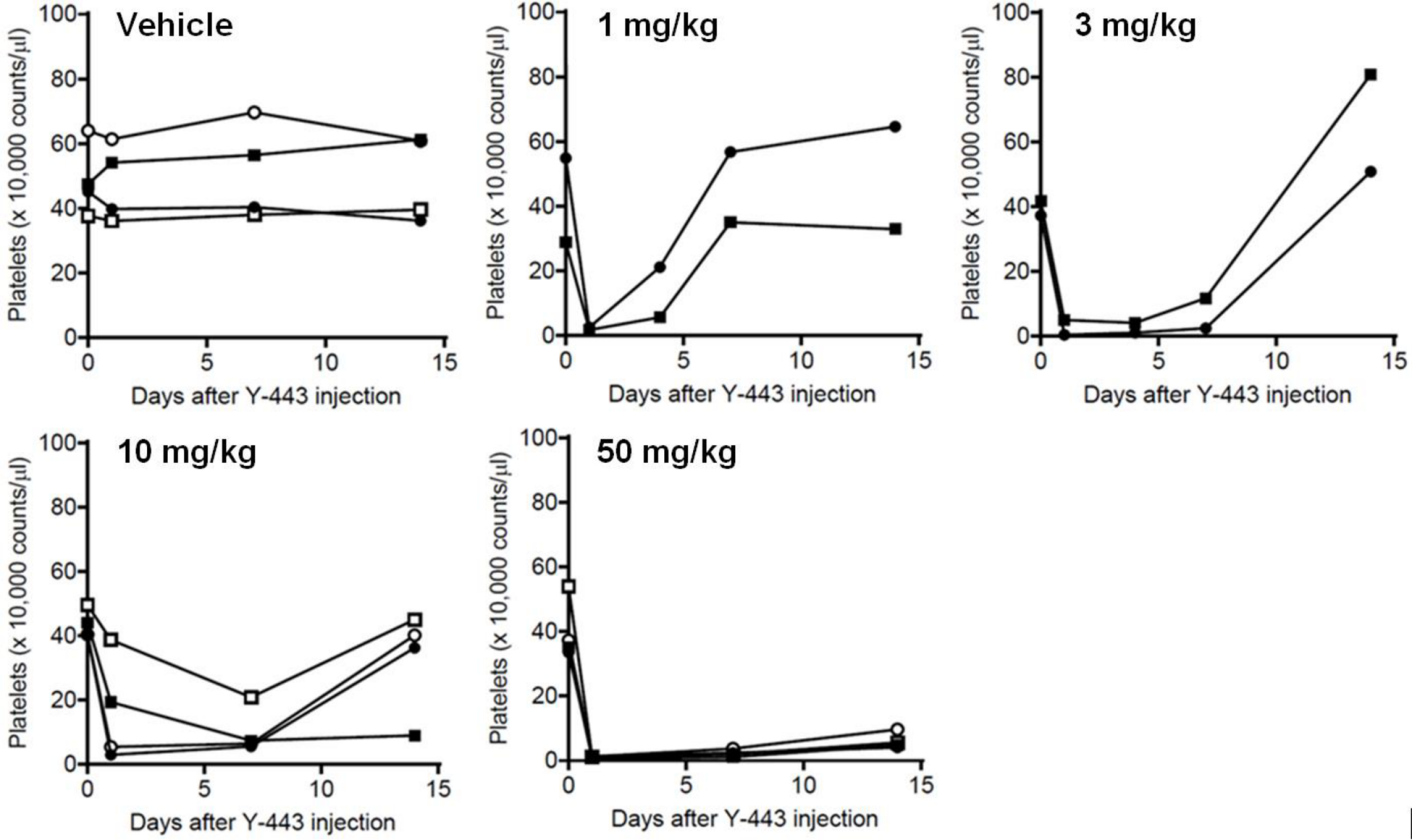
Thrombocytopenia induced by single i.v. administration of Y-443 in Cynos. Platelet count was measured at predose (expressed as day 0) and on days 1, 4, 7 and 14 after the injection of vehicle or Y-443 (1 mg/kg, 3 mg/kg, 10 mg/kg or 50 mg/kg). Each line indicates platelets count of individual monkey. Closed circle and closed square are the result of males, and open circle and open square are the ones of females.

As described above, Y-443 demonstrated an *in vivo* anti-cancer effect suggesting the potential for a therapeutic anti-cancer antibody. Unfortunately, the Y-443 did not cross-react to mouse Nectin-2-ED-Fc; however, Y-443 cross-reacts to Cyno Nectin-2-ED-Fc with a nearly identical affinity to human Nectin-2-ED-Fc (K_D_ = 5.8 nM vs. 4.9 nM, determined by kinetic analysis with Biacore). Therefore, toxicological studies were carried out in Cynos. Single intravenous bolus injection of Y-443 at 10 mg/kg or 50 mg/kg (2 males and 2 females in each group) did not cause death or treatment-related abnormalities as measured by clinical signs including body weights and food consumption for 2 weeks over the observation period (data not shown). However, in the hematological examination, a decrease in platelet count was observed on days 1 and 7 in nearly all animals dosed at 10 mg/kg. Recovery of the platelet counts was observed on day 14 in all monkeys with the exception of one male monkey (Table 1 and Fig 2). At the higher 50 mg/kg dosage, the thrombocytopenia continued for 2 weeks post dosing. The platelet count decreased to less than 10 x 10^4^/μL in almost all animals dosed at 10 and 50 mg/kg ranging from 2 to 42% of pre-treatment values. Decreases in erythrocyte count, hematocrit value and hemoglobin concentration were also observed on days 7 and 14 in one male and all females dosed with 50 mg/kg Y-443. Additionally, the reticulocyte count was found to be increased on days 7 and 14 in most animals dosed with 10 and 50 mg/kg (Table 1), but no changes in leukocytes numbers were observed (data not shown). On day 15, prolongation of bleeding time was observed in 1 male dosed of 10 mg/kg and the both males and one female dosed of 50 mg/kg (Table 2). These changes were considered to be secondary events to thrombocytopenia. Conversely, no treatment-related abnormality was observed in coagulation tests, including prothrombin time (PT), activated partial thromboplastin time (APTT) and plasma fibrinogen in any of the monkeys (Table 2).

**Table 1:** Hematology of Y-443-treated Cynos.

Tested	Animal	Day	Erythrocytes	Hematocrit	Hemoglobin	Platelets	Reticulocytes
article	number		(x10^4^/μL)	(%)	(g/dL)	(x10^4^/μL)	(x10^4^/μL)
Control	1M001	-4	653	41.3	13.1	45.2	2.6
	(male)	1	601	38.0	12.0	39.8	3.0
		7	599	38.4	12.1	40.4	5.4
		14	584	37.6	11.7	36.2	4.7
	1M002	-4	636	40.5	11.7	47.5	1.9
	(male)	1	613	38.7	11.3	54.2	1.8
		7	638	40.1	12.2	56.5	4.5
		14	602	37.5	11.4	61.3	4.2
	1F001	-4	587	40.8	12.3	64.0	2.3
	(female)	1	522	36.3	11.0	61.4	2.1
		7	534	37.5	11.4	69.7	8.5
		14	541	38.2	11.6	60.9	7.0
	1F002	-4	480	37.7	12.2	37.7	6.2
	(female)	1	476	37.1	12.2	36.1	5.2
		7	489	38.0	12.7	38.0	6.4
		14	492	37.8	12.7	39.6	6.4
Y-443	2M001	-4	563	38.3	12.2	40.3	10.1
	(male)	1	533	36.2	11.2	3.0	11.2
10		7	514	34.0	11.2	5.6	23.1
mg/kg		14	548	36.5	11.4	36.3	16.4
	2M002	-4	587	42.2	14.0	44.1	8.8
	(male)	1	553	38.9	13.3	19.4	8.8
		7	527	36.1	12.2	7.4	28.5
		14	549	36.3	12.6	9.0	30.2
	2F001	-4	637	41.2	13.1	40.4	1.9
	(female)	1	581	37.2	11.5	5.5	7.0
		7	566	34.5	11.3	6.5	18.7
		14	569	35.3	11.4	40.2	15.4
	2F002	-4	474	38.3	12.5	49.5	5.7
	(female)	1	478	39.0	12.5	38.7	11.0
		7	475	38.4	12.7	20.9	17.1
		14	482	38.8	12.7	45.0	15.4
Y-443	3M001	-4	663	41.6	13.4	33.5	4.0
	(male)	1	648	39.8	12.9	1.4	5.2
50		7	599	36.1	12.2	2.4	18.6
mg/kg		14	633	38.4	12.5	4.1	13.9
	3M002	-4	619	40.9	12.7	35.0	2.5
	(male)	1	603	39.5	12.1	0.7	3.0
		7	496	32.2	10.0	1.1	15.4
		14	487	32.7	9.9	4.8	24.4
	3F001	-4	570	38.5	12.2	37.3	5.7
	(female)	1	547	36.8	11.6	1.3	5.5
		7	424	29.5	9.3	3.7	57.7
		14	535	37.5	11.5	9.7	14.4
	3F002	-4	683	41.3	11.7	53.9	4.1
	(female)	1	655	38.4	11.0	1.3	6.6
		7	423	23.7	7.2	2.0	36.4
		14	440	27.4	7.7	5.6	41.8

Day 0: the day of Y-443

**Table 2:** Bleeding time, coagulation factors and spleen weight of Y-443-treated Cynos at 2 weeks after dosing.

Tested article	Dose (mg/kg)	Sex	Animal number	Bleeding time day 15 (min)	Coagulation test (day 14)			Spleen weight (% of body weight)
					PT (s)	APTT (s)	Fibrinogen (mg/dl)	
Control		Male	1M001	1.5	9.3	19.1	231	0.17
			1M002	3.5	8.7	18.8	214	0.23
		Female	1F001	1.5	8.7	18.8	239	0.1
			1F002	1.5	9.3	25.9	151	0.06
Y-443	10	Male	2M001	2.5	9.3	20.1	221	0.28
			2M002	5	9.8	19.3	196	0.29
		Female	2F001	1.5	9.4	18.5	253	0.18
			2F002	2	8.2	18	174	0.12
Y-443	50	Male	3M001	6	9.9	20.1	157	0.32
			3M002	17	9.3	19.2	187	0.35
		Female	3F001	3	9	18.5	228	0.25
			3F002	12.5	9.2	17.9	200	0.24

PT: Prothrombin time, APTT: Activated partial thromboplastin time

All Cynos were sacrificed 2 weeks post dosing and subjected to necropsy and histopathological examination. Hemorrhage was observed in several organs (liver, heart, lung, stomach, testis, pancreas, lymph nodes, thymus, thyroids, adipose tissue in abdominal cavity and skeletal muscle) for all of the Cynos dosed of 50 mg/kg. Increased spleen weight was observed in 1 male and 1 female dosed of 10 mg/kg and all the Cynos dosed of 50 mg/kg (Table 2). There were no perceived gender-differences in the aforementioned adverse events. All the symptoms observed in Y-443-treated Cynos, including thrombocytopenia, bleeding tendency, hemorrhage in various organs and splenomegaly, were similar to typical symptom of ITP.

**Fig 3:**
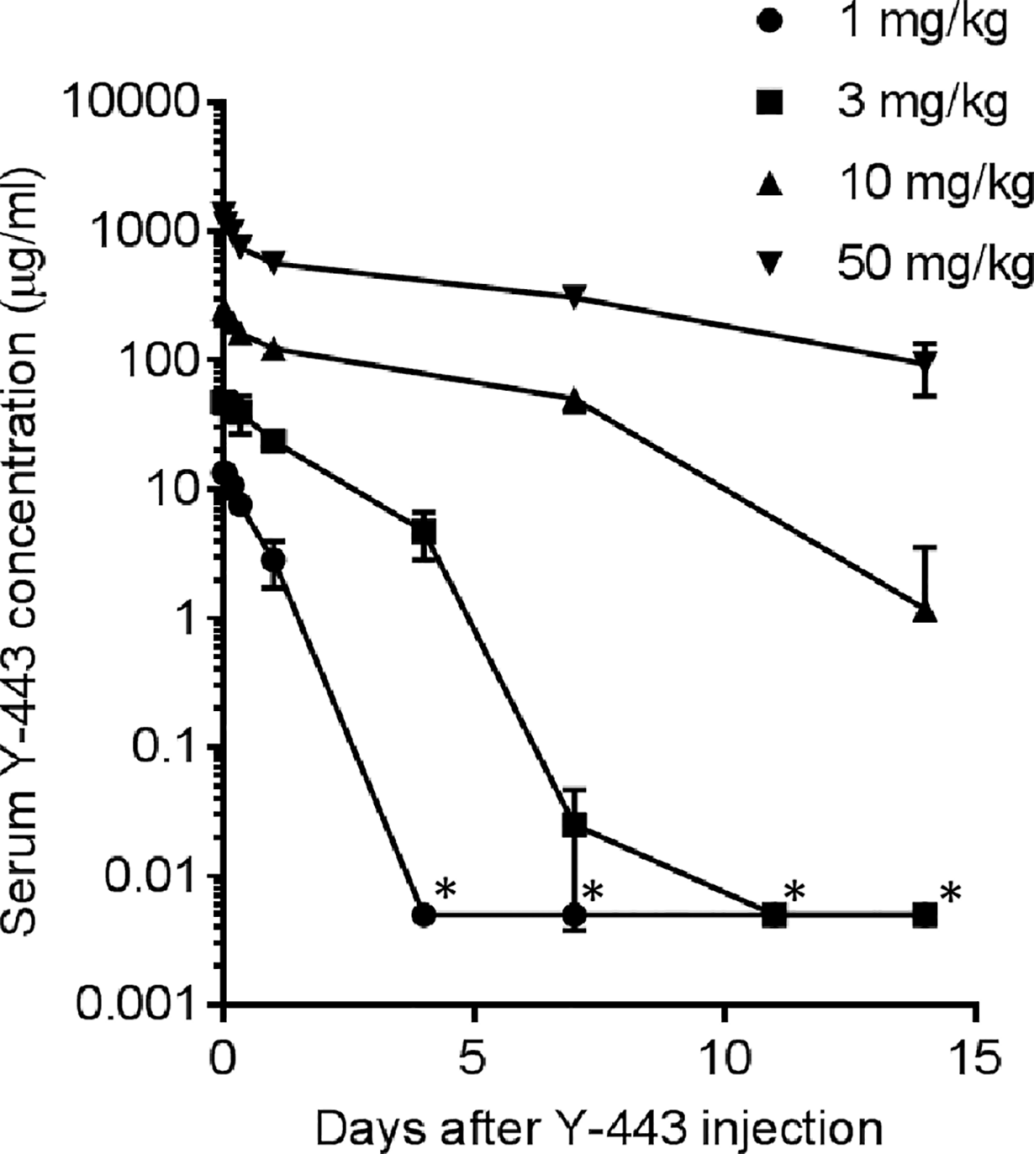
Serum concentration of Y-443 after a single i.v. administration to Cynos. Cyno sera were corrected 0, 1, 4, 8, 24, 168, 264 and 336 hours after a bolus injection of Y-443 at 1 mg/kg and 3 mg/kg (n = 2), or 0, 1, 4, 8, 24, 168 and 336 hours after the injection of Y-443 at 10 mg/kg and 50 mg/kg (n = 4). Y-443 concentrations in the Cyno sera were measured by ELISA. The results are the mean ± S.D. of serum antibody concentration. *: lower concentration than the quantification limit (5 ng/mL).

In order to investigate the dose-dependency of the thrombocytopenia, we included two additional experimental groups, each consisting of two male monkeys treated with bolus injections of either 1 or 3 mg/kg Y-443. Marked decreases in platelet count were reproduced on day 1 in both administration groups (Fig 2). The platelet count recovered earlier in 1 mg/kg group compared to the higher dose groups, although, dose-dependency between 3 and 10 mg/kg groups was insignificant. In toxicokinetics studies, Cynos administered with lower doses of Y-443, especially in the 1 and 3 mg/kg groups, showed accelerated serum clearance of the antibody (Fig 3). Collectively, the results suggest the severity of thrombocytopenia is dependent on the serum concentration of Y-443.

### Fc engineering to mitigate thrombocytopenia

Multiple studies revealed that ITP is triggered by binding of autoantibodies against self-antigens on platelets, and the interaction of the IgG Fc with FcγRI on macrophages drives phagocytosis in the spleen [22–24, 31]. As such, we speculated the possibility of mitigating the Y-443-induced thrombocytopenia through modulating the interaction between the Fc region of antibody and FcγRI. Fortunately, multiple previous studies revealed that mutations of Leu^235^ (Kabat numbering) was capable of weakening the Fc-FcγRI interaction [32–37]. Hence, we generated several Fc mutants of Y-443 and evaluated their binding to recombinant human FcγRs in an ELISA based assay. Furthermore, we also measured the equilibrium dissociation constant (K_D_) against human Nectin-2 by BIACORE to confirm retained target binding activity of the Fc mutant antibodies. We identified two mutants, Y-443 (L235Y) and Y-443 (L235D), both of which exhibited a significant decrease in binding to FcγRI, but retained antigen binding activity relative to the Y-443 parental antibody (Table 3). The Y-443 (L235Y) mutant also exhibited moderate decrease in binding to FcγRIIa and FcγRIIb, and 30-fold reduction in binding to FcγRIIIa (158F). Alternatively, the Y-443 (L235D) mutant displayed a lesser change in binding to FcγRIIa and FcγRIIb, but only a 5-fold reduction in binding to FcγRIIIa was observed. Therefore, the Y-443 (L235D) mutant was selected for future studies as it demonstrated the most desirable features.

**Table 3:** Binding activity of Y-443 and Fc-mutated antibodies to human Nectin-2 and human FcγRs.

	K_D_ values against human Nectin-2	*Relative binding activity to human FcγRs (%)			
		FcγRI	FcγRIIa	FcγRIIb	FcγRIIIa (158F)
Y-443	3.2 nM	100	100	100	100
Y-443 (L235Y)	3.3 nM	0.017	26	35	3.4
Y-443 (L235D)	1.5 nM	0.074	64	120	22
Y-634	3.2 nM	0.056	46	81	300

*Relative binding activity (%) = EC_50_ (Y-443) / EC_50_ (sample) x 100

**Fig 4:**
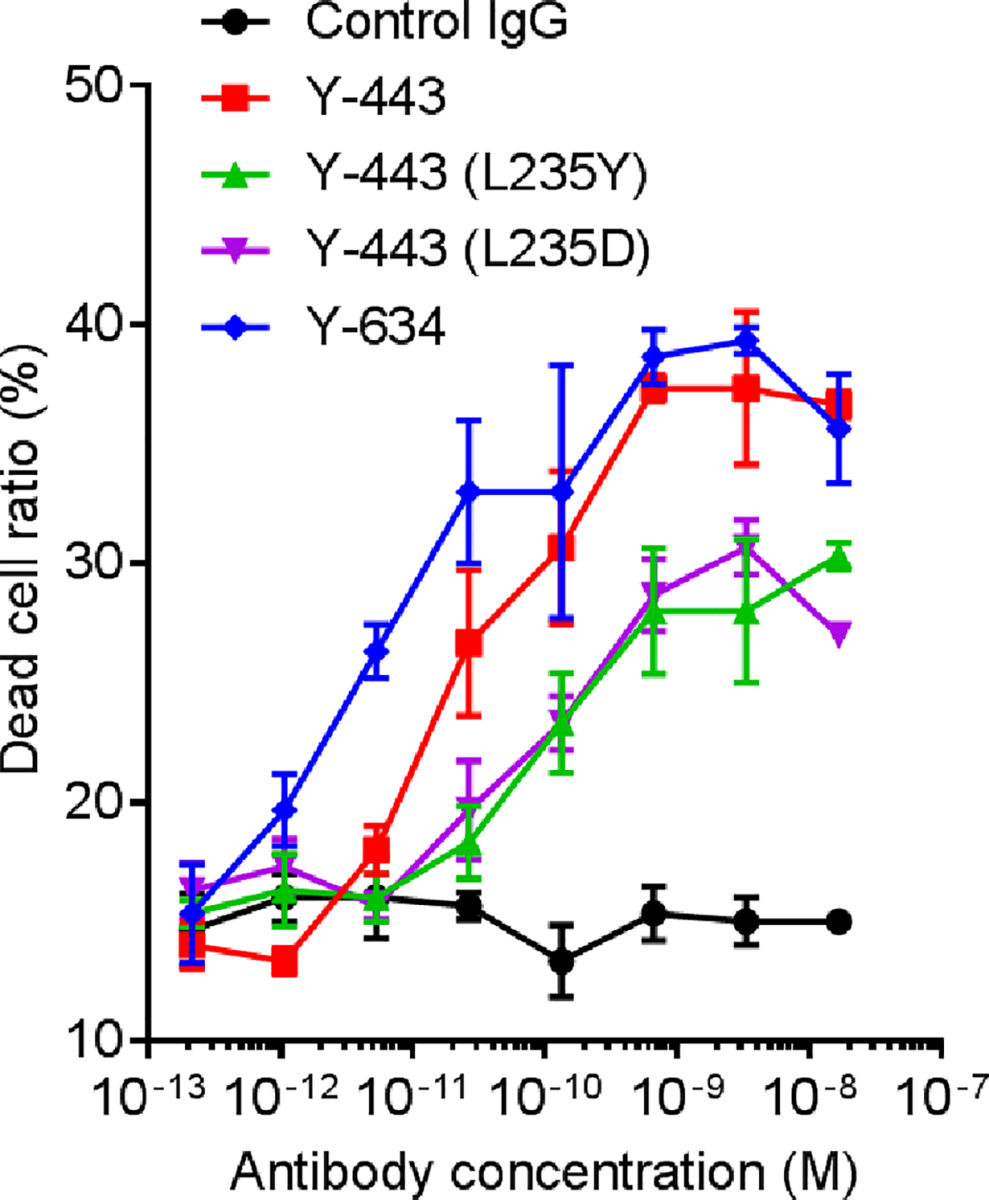
ADCC of Y-443 and Fc-mutated antibodies against MDA-MB-231 cells. MDA-MB-231 cells pre-labeled with Calcein AM were incubated with different concentrations of Y-443 or the Fc mutants, followed by addition of PBMC effector cells at a ratio of 1:50. The cell mixture was incubated for 4 hours at 37°C, and Calcein AM intensity in cells was detected by Acumen eX3 (TTP labtech). The results are the mean ± S.D. of dead cell ratio.

In consideration that ADCC is the primary mechanism for anti-tumor effect of Y-443 [21], the ADCC of the Y-443 mutants was evaluated. As predicted by the decreased affinity to FcγRIIIa, ADCC of Y-443 (L235D) against MDA-MB-231 breast cancer cells was 10-fold lower than its parent antibody, Y-443 (Fig 4).

To rescue the decreased ADCC potency, we prepared a defucosylated variant of the Y-443 (L235D) mutant. Removal of the L-fucose from N-glycan linked to asparagine (Asn) residue at Kabat position 297 was accomplished by stably expressing a plasmid encoding Y-443 (L235D) antibody gene in POTELLIGENT^®^ Cells. The recombinant defucosylated Y-443 (L235D) antibody, termed Y-634, was purified from the culture supernatant as described in Materials and Methods. The L-fucose content of Y-634-derived N-glycan was undetectable compared to those of Y-443 and Y-443 (L235D). The Nectin-2 binding affinity of Y-634 was identical to Y-443 and Y-443 (L235D) (Table 3). Moreover, the ADCC potency of Y-634 was comparable to Y-443 (Fig 4). Thus, we demonstrate successful development of Y-634, a defucosylated Y-443 (L235D) mutant, with significantly reduced binding to FcγRI while maintaining both Nectin-2 binding activity and ADCC.

### Toxicological studies of Y-634 in Cynos

**Fig 5:**
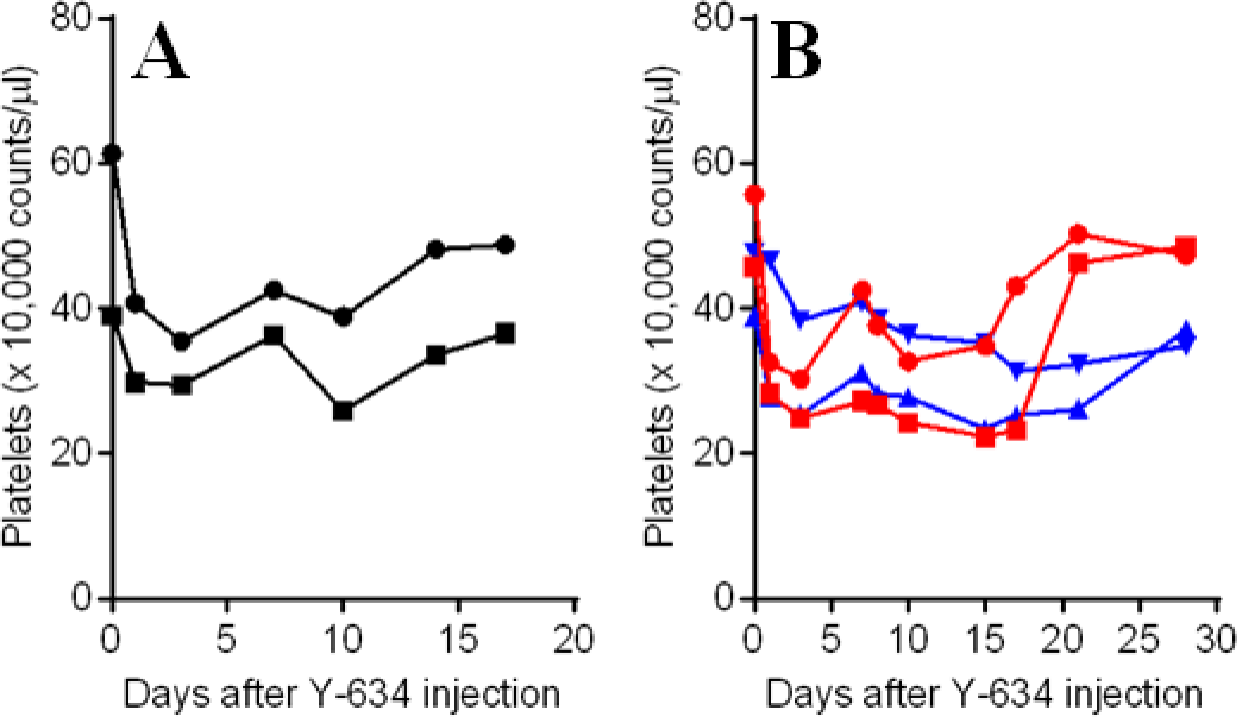
Thrombocytopenia induced by single and repeated i.v. administration of Y-634 in Cynos. For the single dose group (A), platelet count was measured in Cynos treated with 10 mg/kg of Y-634 at predose (expressed as day 0) and on days 1, 3, 7, 10, 14 and 17. For the weekly repeated intravenous administration (B) of 3 mg/kg (red lines) or 10 mg/kg (blue lines) of Y-634 dosed on days 0, 7 and 14, platelet count was measured in Cynos at predose (expressed as day 0) and on days 1, 3, 7 (pre 2nd dose), 8, 10, 15, 17, 21 and 28. Each line indicates platelet count of individual monkey.

To investigate the pharmacovigilance, Y-634 was subjected to toxicological studies in Cynos. The effect of a single intravenous bolus injection (10 mg/kg) of Y-634 on plasma platelets was investigated in female monkeys (n = 2). Unfortunately, platelet counts were decreased to 30 x 10^4^ and 41 x 10^4^/μL level on day 1, although, there were no major changes in hematological values (S1 Table). Nevertheless, the degree of thrombocytopenia was milder than those of Y-443, and the platelet level was maintained between 29 x 10^4^ and 49 x 10^4^/μL over the two week observation period. The platelet levels never reached below 10 x 10^4^/μL (or one third of the normal Cyno’s platelet value), which for comparison, is assumed to be a critical level in human thrombocytopenia patients for observed prolonged bleeding time in terms of safety (CTCAE v3.0; https://ctep.cancer.gov/protocoldevelopment/electronic_applications/ctc.htm) (Fig 5 and S1 Table). In a repeated intravenous administration study in Cynos, dosed at either 3 mg/kg/week or 10 mg/kg/week (n = 2), platelet counts were maintained above 22 x 10^4^/μL for 4 weeks after the 1st dosing of Y-634 in the both groups and further reduction by repeated dosing was not observed. Necropsy or histopathological examination showed no notable changes which had been observed in the study of Y-443 (data not shown). Ultimately, the severe thrombocytopenia induced by Y-443 was markedly reduced through Fc engineering; however, complete elimination of thrombocytopenia was not observed.

### Tumor growth inhibition by Y-634 against MDA-MB-231 in a mouse subcutaneous xenograft model

**Fig 6:**
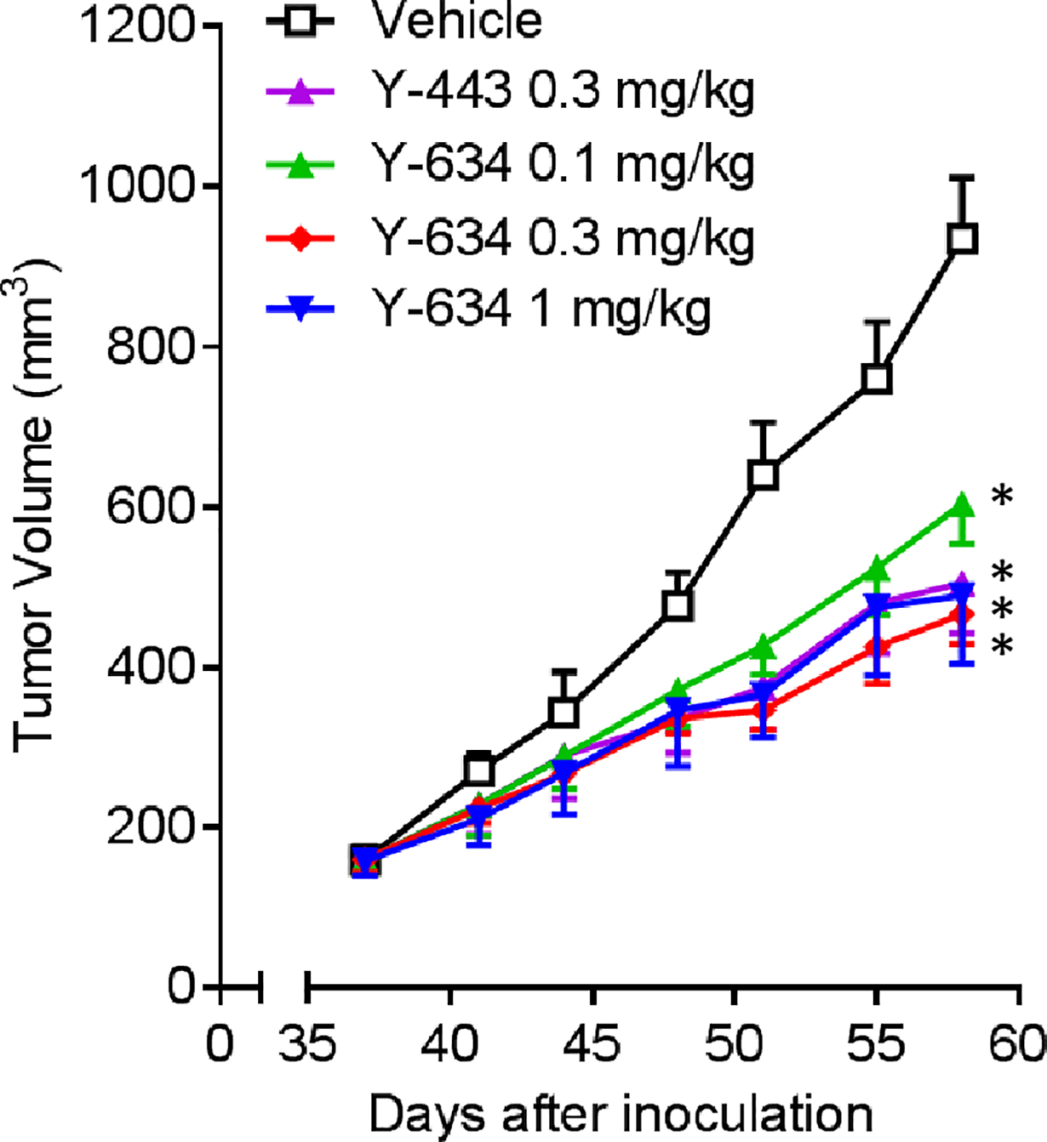
*In vivo* anti-tumor effect of Y-634 in a mouse subcutaneous xenograft model with MDA-MB-231 cells. Anti-tumor effect of Y-634 was compared with its parental antibody, Y-443, in a MDA-MB-231 mouse subcutaneous xenograft model. On days 37, 44 and 51 after MDA-MB-231 cell inoculation, Y-443 at a dose of 0.3 mg/kg, Y-634 at a dose of 0.1, 0.3 and 1 mg/kg or vehicle was intravenously administered (n = 5). The results are the mean ± S.D. of tumor volume. **: p < 0.001 versus the tumor volume treated with vehicle by one-tailed Williams’ test.

Y-634 showed similar ADCC activity compared to the parent antibody, Y-443, and demonstrated partially alleviated thrombocytopenia in Cynos (Fig 4). In the final experiment, we evaluated the anti-tumor effect of Y-634 in a mouse subcutaneous xenograft model of MDA-MB-231 breast cancer cells. Weekly administrations of Y-634 showed significant anti-tumor effect at 0.1 mg/kg and equivalent efficacy to Y-443 at 0.3 mg/kg (Fig 6). Altogether, these results suggest that Y-634 retains strong anti-tumor effect with significantly reduced thrombocytopenia.

## Discussion

In our previous study, we demonstrated that Nectin-2 is over-expressed in both breast and ovarian cancers [21]. We subsequently developed an anti-Nectin-2 fully human mAb, Y-443, which has an *in vivo* anti-tumor effect on OV-90 and MDA-MB-231 xenograft models through an ADCC mechanism [21]. Since the Y-443 cross-reacted to Cyno Nectin-2 with equivalent K_D_ to human Nectin-2 (4.9 nM and 5.8 nM, respectively), we conducted toxicological studies in Cynos. In a single dose study, intravenous administration of Y-443 (10 and 50 mg/kg) caused severe thrombocytopenia with prolonged bleeding time in all the animals (Table 1, Table 2 and Fig 2), despite that clinical signs and other hematological remained normal. Interestingly, the Nectin-2 expression was observed only in liver and testes [21], with negligible expression on human platelets (data not shown), hence, the adverse events observed in Cynos were unexpected. Here, Y-443 marginally detected Nectin-2 on Cyno platelets (data not shown), and furthermore, did not induce aggregation of monkey platelets at 100 μg/mL in *in vitro* (data not shown). Moreover, single intravenous administration of Y-443 at 10 and 50 mg/kg gave no meaningful changes in Cyno coagulation test (Table 2). The splenomegaly observed in those monkeys correlated to the activated phagocytic function of the spleen, which seemed to cause disposal of platelet-Y-443 complex resulting in thrombocytopenia and faster serum clearance of the antibody in the lower dose groups. The activated phagocytic function could conceivably result in the observed anemia of the dosed animals (Table 1). The platelet count recovered within the two week post-treatment observation period in the lower dosing groups (1, 3 or 10 mg/kg), but not the 50 mg/kg dosing group (Fig 2). The reversibility of thrombocytopenia observed in the 1, 3 and 10 mg/kg dosing groups and increased reticulocyte count (days 7 and 14 in the 10 and 50 mg/kg dosing groups) suggest that the platelet production by the bone-marrow was unimpaired by the antibody treatment. The symptoms observed in Y-443-treated Cynos were similar to symptoms of thrombocytopenia (bleeding tendency, hemorrhage in organs, and splenomegaly), which were likely to be target mediated toxicity. ITP is occasionally caused by auto-antibodies against self-antigens on platelets [22–24]. Consequently, the presented data suggests that Y-443 might act like an autoantibody in Cynos by inducing ITP-like symptoms presumably causing phagocytosis of Y-443-bound platelets by macrophages in the spleen.

Previous reports suggested that disruption of the interaction of autoantibodies and FcγRI is useful in preventing phagocytosis of the antibody-coated platelets by macrophages [31]. Additionally, multiple studies utilizing Fc engineering demonstrated that the mutations of Leu^235^ were capable of diminishing the Fc-FcγRI interaction [32–37]. We have shown that ADCC was the main mechanism of tumor regression of anti-Nectin-2 mAb, Y-443, for which binding of the Fc to FcγRIIIa is essential to maintain ADCC. Therefore, we applied Fc engineering to reduce the binding affinity of the Y-443 Fc to FcγRI while maintaining its binding to FcγRIIIa. Mutation of Leu^235^ resulted in the L235D mutant, Y-443 (L235D), which showed markedly decreased binding to human FcγRI with only a mild reduction (4.5-times) in binding to human FcγRIIIa, as compared to the parental Y-443 antibody. However, as predicted, Y-443 (L235D) showed a 10-fold weaker ADCC against MDA-MB-231 breast cancer cells (Fig 4). Nevertheless, it has been reported that the lack of L-fucose on N-linked oligosaccharide on Asn^297^ of human IgG_1_ antibodies can enhance their binding affinity to FcγRIIIa and consequently the ADCC. Therefore, we sought to recover the ADCC of Y-443 (L235D) mutant through defucosylation of the N-glycan on the Asn^297^ using the POTELLIGENT^®^ technology (see Materials and methods) [39, 40]. The defucosylated Y-443 (L235D) variant, Y-634, showed potent ADCC, comparable to that of the Y-443 antibody while further maintaining the diminished binding affinity to human FcγRI as observed with the Y-443 (L235D) mutant (Fig 4 and Table 3). Thus, Y-634 demonstrated diminished thrombocytopenia in the Cynos both upon single and repeated dose administration, but also maintained the anti-tumor effect against breast and ovarian cancer cells in mouse xenograft models (Fig 5, Fig 6 and S1 Table). Although we haven’t evaluated the binding activities of Y-443 mutants to Cyno FcγRs, it has been reported that human IgG_1_ bound to Cyno FcγRI and FcγRIIIa with similar binding affinities to human FcγRI and FcγRIII, respectively [41]. In addition, the amino acid sequence of the human IgG_1_ binding sites on Cyno FcγRI and FcγRIIIa are nearly identical to those in human FcγRI and FcγRIIIa, respectively [42, 43]. Therefore, the improved features in Y-634 presumably reflect the binding activities to Cyno FcγRI and FcγRIIIa.

In summary, we demonstrated a potential strategy to overcome ITP-like toxicity of therapeutic antibodies by combining a single amino acid mutation in Fc region and glyco-engineering. Our findings may help facilitate the development of antibody therapeutics through a strategy to reduce observed side effects like thrombocytopenia while maintaining antibody efficacy.

## Supporting information

S1 Table Hematology of Y-634-treated Cynos. (DOCX)
