# Supplementary material for: Fc engineering of anti-Nectin-2 antibody improved thrombocytopenic adverse event in monkey
Source: PLoS One. 2018 May 3;13(5):e0196422. doi: 10.1371/journal.pone.0196422 (PMC5933732; doi:10.1371/journal.pone.0196422)
Supplement: S1 Table — (DOCX) [file pone.0196422.s001.docx]

# Supporting information

**S1 Table. Hematology of Y-634-treated Cynos.**

| Tested | Animal | Day | Erythrocytes | Hematocrit | Hemoglobin | Platelets | Reticulocytes |
| --- | --- | --- | --- | --- | --- | --- | --- |
| article | number |  | (x10^4^/µL) | (%) | (g/dL) | (x10^4^/µL) | (x10^4^/µL) |
| Y-634 | 1F001 | -4 | 608 | 39.2 | 10.9 | 61.4 | 2.4 |
|  | (female) | 1 | 620 | 39.5 | 11.1 | 40.8 | 1.2 |
| 10 |  | 3 | 578 | 37.3 | 10.3 | 35.5 | 1.7 |
| mg/kg |  | 7 | 581 | 37.8 | 10.5 | 42.6 | 4.6 |
|  |  | 10 | 577 | 37.3 | 10.4 | 38.9 | 4.0 |
|  |  | 14 | 573 | 37.4 | 10.4 | 48.2 | 4.0 |
|  |  | 17 | 575 | 37.7 | 10.4 | 48.8 | 2.9 |
|  | 1F002 | -4 | 574 | 39.1 | 11.3 | 39.0 | 4 |
|  | (female) | 1 | 548 | 37.3 | 11.1 | 29.8 | 3.8 |
|  |  | 3 | 532 | 36.1 | 10.5 | 29.5 | 4.8 |
|  |  | 7 | 555 | 37.7 | 11.1 | 36.3 | 6.1 |
|  |  | 10 | 554 | 37.4 | 11.0 | 26.0 | 2.2 |
|  |  | 14 | 549 | 37.6 | 11.0 | 33.7 | 3.8 |
|  |  | 17 | 527 | 36.1 | 10.6 | 36.6 | 4.7 |

Day 0: the day of Y-634 injection
